# USP18 restricts PRRSV growth through alteration of nuclear translocation of NF-κB p65 and p50 in MARC-145 cells

**DOI:** 10.1016/j.virusres.2012.07.002

**Published:** 2012-10

**Authors:** Dequan Xu, Simon G. Lillico, Mark W. Barnett, Christopher B.A. Whitelaw, Alan L. Archibald, Tahar Ait-Ali

**Affiliations:** a Key Laboratory of Swine Genetics and Breeding of Ministry of Agriculture, and Key Lab of Agricultural Animal Genetics, Breeding and Reproduction of Ministry of Education, Huazhong Agricultural University, Wuhan 430070, China; b The Roslin Institute and Royal (Dick) School of Veterinary Studies, Easter Bush, EH25 9RG Midlothian, UK

**Keywords:** USP18, NF-κB, MARC-145, PRRSV, Pig

## Abstract

Although the functions of porcine respiratory and reproductive syndrome virus (PRRSV) proteins are increasingly understood, the roles of host factors in modifying infection are less well understood. Growing evidence places deubiquitination at the core of a multitude of regulatory processes, ranging from cell growth to innate immune response and health, such as cancer, degenerative and infectious diseases. This report provides further information on the functional role of the porcine ubiquitin-specific peptidase 18 (USP18) during innate immune responses to PRRSV. We have shown that constitutive overexpression of the porcine USP18 in MARC-145 cells restricts PRRSV growth, at least in part via early activation of NF-κB. Viral growth of PRRSV may be perturbed by increasing and decreasing nuclear translocation of p65 and p50, respectively. Our data highlight USP18 as a host restriction factor during innate immune response to PRRSV.

Porcine reproductive and respiratory syndrome virus (PRRSV) is an enveloped RNA virus in the family *Arteriviridae* that causes highly significant economic losses to the swine industry worldwide (Neumann et al., 2005). The ubiquitin-specific peptidase 18 encoded by the USP18 gene has been shown to be a negative regulator of type-I interferon signaling (IFN) in both murine and porcine systems (Malakhova et al., 2003; Ait-Ali et al., 2009). More recently genetic and biochemical evidence has suggested important roles for USP18 in immune function via ISG15 protease-dependent and -independent fashions (Liu et al., 2005; Sun, 2008; Sun et al., 2010; Chen et al., 2011). Mouse embryonic fibroblasts (MEF) and bone marrow-derived macrophages from USP18−/− mice showed restricted lymphocytic choriomeningitis virus (LCMV) replication (Ritchie et al., 2004). In addition, the USP18−/− mice were able to restrict the growth of *Salmonella typhimurium* more efficiently than wild-type mice (Zou et al., 2007). The objective of this study was to examine USP18 as a restriction factor during PRRSV infection in a stably transfected cell line and explore possible mechanisms of USP18 restriction of PRRSV infection. It should be noted that neither mice, nor murine cell lines are susceptible to PRRSV infection.

The full length USP18 cDNA (AF134195) harbored a Cys-box and a His-box (Supplemental data 1). The Cys-box has putative transmembrane helices and a conserved putative active-site cysteine at cys64, which is essential for the catalytic property of USP18 (Quesada et al., 2004). The mutant version of USP18 (USP18DN) lacks the Cys-box (Supplemental data 1) and is thus unlikely to retain any significant proteolytic activity as demonstrated previously (Ait-Ali et al., 2009). For functional analysis, USP18 and USP18DN cDNAs were PCR-amplified from pcDNA3.1D/V5-His-USP18 and pcDNA3.1D/V5-His-USP18mut (Ait-Ali et al., 2009) with forward primer TAACCGGTATAGGGAGACCCAAGC and reverse primer GATGTACACGCGTAGAATCGAGACC. PCR products were cloned into the pLenti6V5-D-TOPO expression vector (Invitrogen, UK) to generate the expression constructs USP18 and USP18DN tagged with V5-epitope. The integrity of the fusion constructs was confirmed by sequencing.

For lentiviral production, 293 T cells were plated at 1 × 10^5^/cm^2^ in T25 flasks in complete medium (DMEM, glutamax, 10% FCS, NEAA). Twenty hours postplating, transfection mix was prepared by sequential addition as follows: Opti-MEM (Invitrogen) (145 μl), psPAX2 2 μg, VSV-G 1 μg, expression construct 1.5 μg, Fugene HD (Roche) 17 μl. After 20 min at room temperature the transfection mixture was applied to the culture medium on cells. The medium was changed 14–16 h later. The conditioned medium was collected after another 24–48 h, filtered through a 0.45 μm PVDF filter (Millipore), aliquoted and stored at −80 °C.

To assay for lentiviral titer, D17 cells were seeded in a 12-well plate at 6 × 10^4^/well one day prior to transduction. Virus stock was serially diluted (10^−1^–10^−6^) in complete medium containing polybene (8 μg/mL), then 500 μl of dilutions added to the D17 cells, followed 3 h later with a further 1 mL of complete medium. After overnight incubation the medium was aspirated and replaced with 1 mL of complete culture medium containing 15 μg/mL Blasticidin. The medium was replaced with fresh medium containing antibiotic every 3–4 d. Once all cells had died in mock-transduced wells (10–12 days) D17 colonies were counted.

MARC-145 cells, which are derived from African green monkey kidney, and which are used extensively in PRRSV-host cell interaction studies, were transduced with lentivirus expressing USP18 or USP18DN under the control of a constitutive CMV promoter. MARC-145 cells were seeded in a 24-well plate with complete medium. Following overnight incubation medium was aspirated and replaced with virus dilutions in complete medium. After a further 24 h the medium containing virus was removed and replaced with complete culture medium containing 2 μg/mL blasticidin for selection of recombinant USP18 and USP18DN cells.

The accumulation of transcripts encoded by the transgene(s) was measured by semi-quantitative RT-PCR and showed that USP18 and USP18DN mRNA were exclusively expressed in USP18 and USP18DN cells, respectively, when compared to MARC-145 control cells (Fig. 1A). In contrast, USP18-V5 and USP18DN-V5 proteins were only detected in the presence of a cell-permeable proteosome degradation inhibitor MG132 as described earlier by Ait-Ali et al. (2009) (Fig. 1B). This result suggests that the level of both proteins is tightly regulated, at least in part by the proteosome degradation machinery.

USP18 has been associated with the innate immune response to pathogens in multiple organisms (Liu et al., 2005). To examine the biological function of porcine USP18 as a restriction factor during viral infection, 10^4^ recombinant MARC-145 cells were subjected to PRRSV infection with the MARC-145-adapted PRRSV Olot/91 strain at multiplicity of infection (moi) of 0.5 (Plana Duran et al., 1997). Fig. 2A and B shows that the growth of PRRSV Olot/91 was substantially restricted in recombinant MARC-145 cells expressing USP18 cells in contrast to control cells or recombinant MARC-145 cells expressing USP18DN over 72 h of viral infection. Taken together these results indicate that constitutive over-expression of the functional USP18 in MARC-145 cells affects PRRSV growth. We reasoned that the de-ubiquitination activity of USP18 could affect a wide range of early innate immune signaling mechanisms. NF-κB p105/p50 (NFκB1) and p65 (RelA) belong to a family of inducible transcription factors involving pathogen- or cytokine-induced immune and inflammatory responses. Classical NF-κB exists as heterodimers consisting of a 50-kDa subunit (p50) and a 65-kDa subunit (p65) (Ghosh et al., 1998). Many viruses encode proteins that activate or modulate NF-κB signaling pathways for their own advantage (Santoro et al., 2003). For PRRSV, experimental evidences suggest that the nucleocapsid N protein and nsp2 protein may be involved in NF-κB activation (Luo et al., 2008; Lee and Kleiboeker, 2005; Fang et al., 2012). Thus, we tested if USP18 could affect the cellular translocation of NF-κB p65 and p50 24 h post infection with PRRSV Olot/91 using immunolocalization and confocal microscopy (Fig. 3A and B). We found that p50 and p65 cellular distribution was affected only in PRRSV-infected USP18 and USP18DN cells. NF-κB p65 translocated more readily to the nucleus of infected cells expressing USP18 and to some extent USP18DN than in control cells. In contrast NF-κB p50 translocated to the cytosol regardless of whether the infected cells expressed the USP18 or USP18DN transgene. Taken together these data suggest that the restricted growth of PRRSV in USP18 cells could operate by altering the cellular distribution of p65 and p50 early during PRRSV infection and earlier than in infected control cells (Fig. 3A). Previous work has demonstrated that PRRSV activates NF-κB in MARC-145 cells and macrophages late during infection (36–48 hpi) (Lee and Kleiboeker, 2005). The PRRSV N protein, a late viral protein, has been proposed to be responsible for this NF-κB activation in a dose-dependent manner (Sun et al., 2012). Therefore it is tempting to suggest that early induction of NF-κB (24 hpi) mediated by USP18 during PRRSV infection, via increasing and decreasing nuclear translocation of p65 and p50, respectively, is detrimental for viral growth. Further work is needed to investigate the role of USP18 as a viral growth restrictor during early innate immune response in MARC-145 cells and alveolar macrophages.

## Figures and Tables

**Fig. 1:**
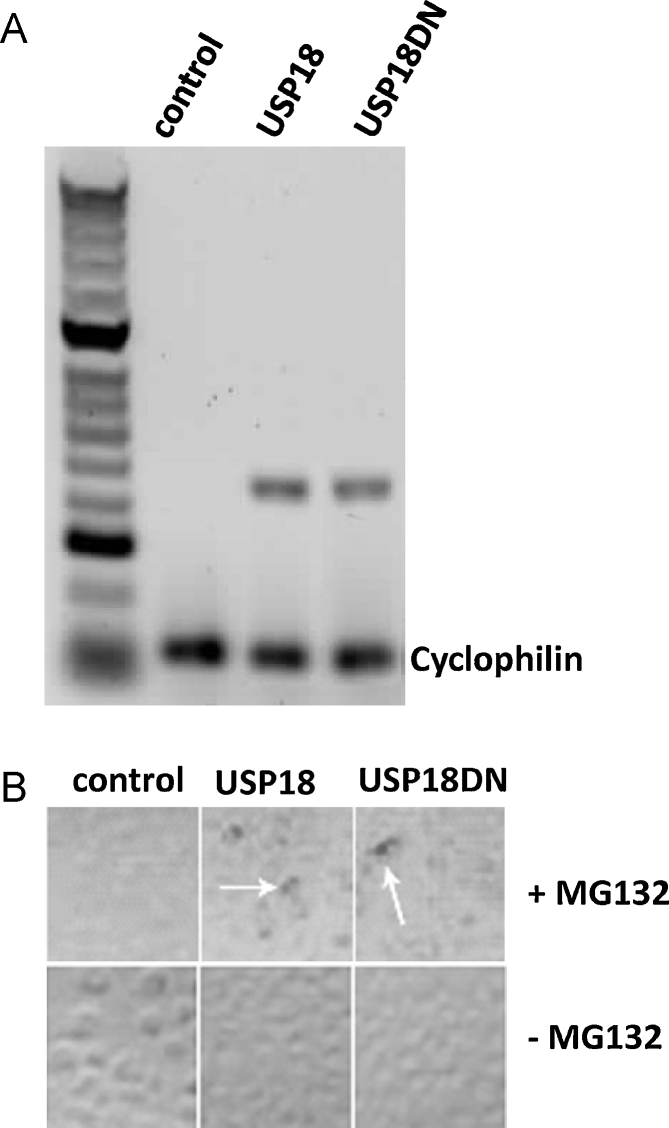
Transcript accumulation of transgenes and immunodetection in recombinant in MARC-145 cell lines. (A) RT-PCR analysis. The RT-PCR reactions are carried out with USP18/USP18DN and V5 specific primers using QIAGEN OneStep RT-PCR kit. RT-PCR amplification of cyclophilin transcripts was used as an internal control. (B) Immunoperoxidase assay. MARC-145, MARC-145-USP18 and MARC-145-USP18DN cells were seeded in a 96-well plate with complete medium. Following overnight incubation cells were either incubated with 10-μM MG132 (+MG132) or mock-treated (−MG132) for 24-h prior to immunodetection which was performed using anti-V5 antibody (Invitrogen) and horseradish peroxidase conjugated rabbit anti mouse (Dako, UK). White arrows denote the expression of USP18-V5/USP18DN-V5 recombinant proteins. M, HyperLadder™ II; Control, MARC-145 cells; USP18, MARC-145-USP18 cells; USP18DN, MARC-145-USP18DN cells.

**Fig. 2:**
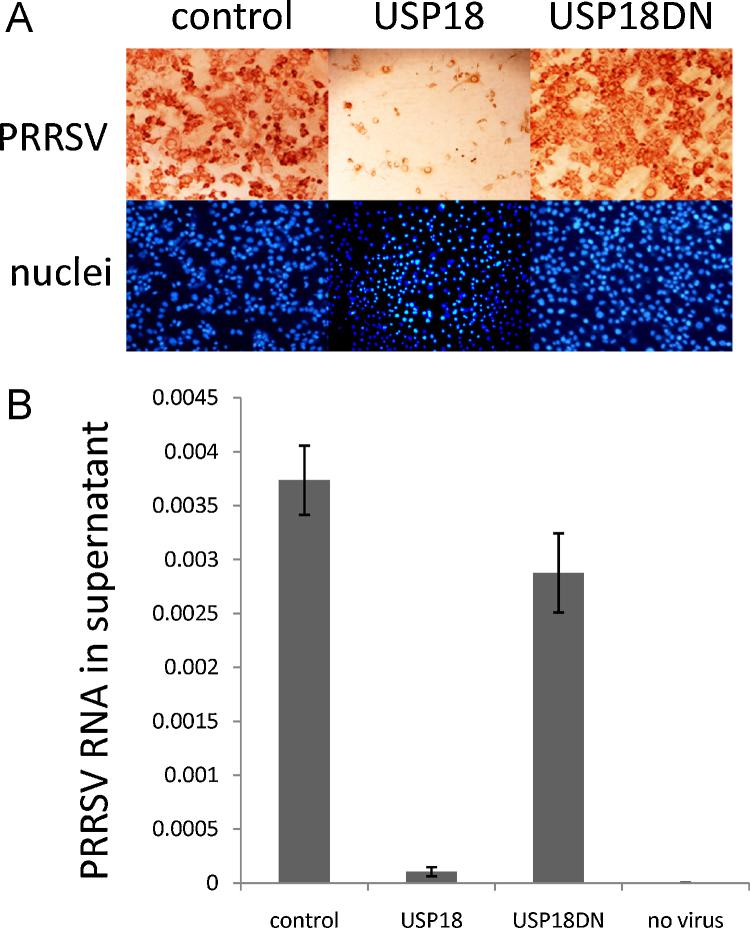
Effect of USP18 and USP18DN on PRRSV Infection in MARC-145 cells. MARC-145 (control), USP18 and USP18DN cells were seeded in a 96-well plate (10^4^/well) with complete medium. Following overnight incubation, cells were infected with a PRRSV Olot/91 strain at a multiplicity of infection of 0.5 for 1-h. The medium was subsequently replaced with fresh medium and cells were incubated for a further 72 h. (A) Cells were stained with PRRSV N protein specific MAb SDOW17 (Rural Technology, USA), and horseradish peroxidase conjugated rabbit anti-mouse polyclonal Ab (Dako, UK) was used as a secondary antibody. The cell nucleus was stained with DAPI (4′,6-diamidino-2-phenylindole; blue fluorescence). Pictures were taken with an Axiovert 25 (Zeiss) microscope. (B), medium from (A) were subjected to RNA isolation using Qiamp viral RNA mini kit (Qiagen, Hilden, Germany) followed by NextGen Real-Time RT-PCR Target Specific Reagents for the detection of PRRS viral RNA (Tetracore, Rockville, USA) according to manufacturer instructions (*n* = 4) and run on a Lightcyler 480 II (Roche Applied Science, USA) in parallel with a series of 10-fold dilutions of PRRSV Olot/91 viral suspension to establish the standard curve. Data are expressed relative to the standard curve. No virus, negative control.

**Fig. 3:**
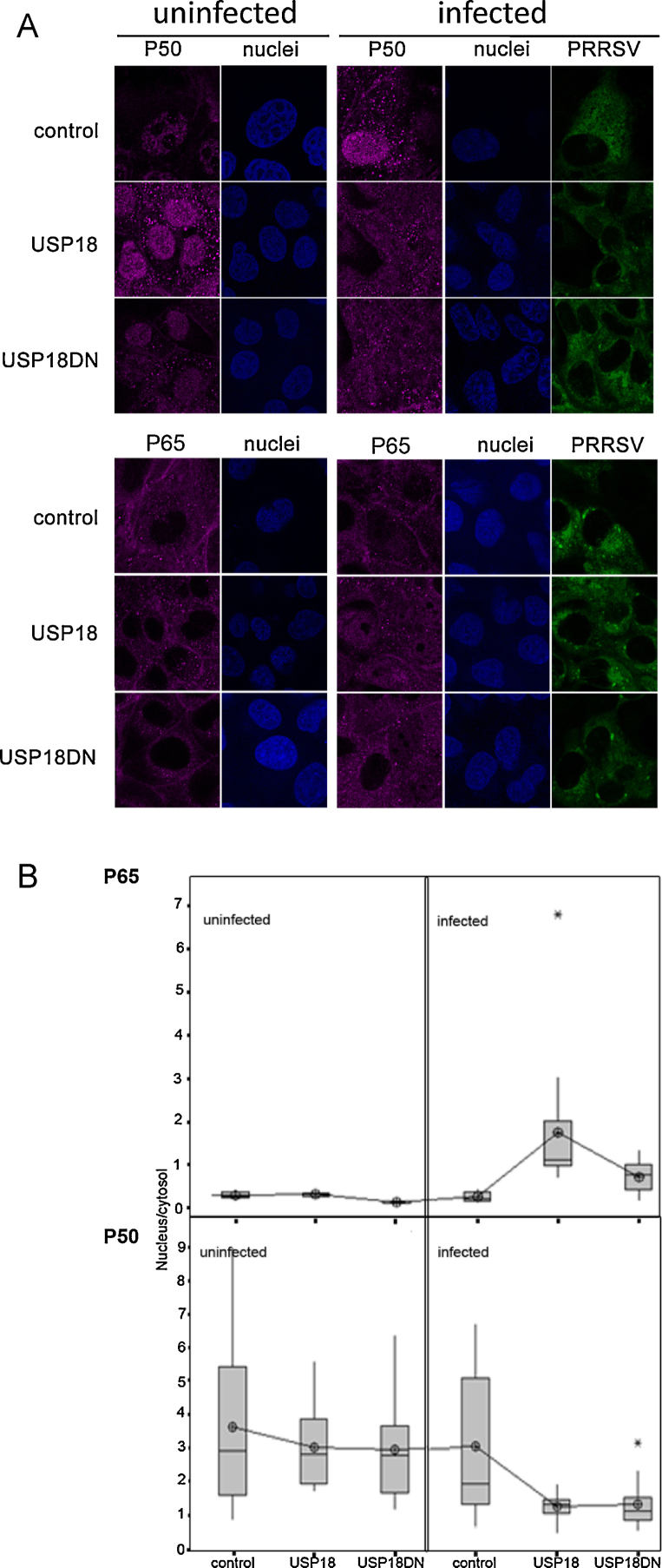
Immunolocalization of NF-κB p65 and p50. MARC-145 (control), USP18 and USP18DN cells were seeded on coverslips in a 48-well plate (10^5^/well) with complete medium. Following overnight incubation, cells were infected with PRRSV Olot/91 strain at a multiplicity of infection of 2 and incubated for 1 h. Infection medium was subsequently replaced with fresh growth medium and cells were incubated for a further 24 h. Uninfected cells were treated similarly as above without addition of virus. Cells were permeabilized and stained with rabbit anti- NF-κB p105/p50 (ab7971) or anti-NF-κB p65 (ab7970) antibodies at dilution 1/100 (ABcam, UK) and the Alexa Fluor 467-conjugated goat anti-rabbit antibody (Life Technologies, UK) was used as the secondary antibody at dilution 1/100. The FITC conjugated SDOW17 (Rural Technologies, USA) was used to detect N protein expression. Stained cells mounted using Vectashield medium containing DAPI (Vector Laboratories, UK). Confocal images were obtained using a Zeiss LSM710 inverted confocal microscope. Images were analyzed using Zen2010 software (Carl Zeiss, UK). (A) Immunolocalization of p65 and p50 in infected and non-infected cells. (B) Image processing and analysis of signal ratios nucleus/cytosol from (A) for P65 and P50 channels (*n* = 18–30 cells) using ImageJ 1.45s (NIH, USA) and statistical analysis by one-way ANOVA (Minitab 16).
