# Supplementary figures and images for: USP18 restricts PRRSV growth through alteration of nuclear translocation of NF-κB p65 and p50 in MARC-145 cells
Source: Virus Res. 2012 Oct;169(1):264–7. doi: 10.1016/j.virusres.2012.07.002 (PMC3501733; doi:10.1016/j.virusres.2012.07.002)

## Slide 1
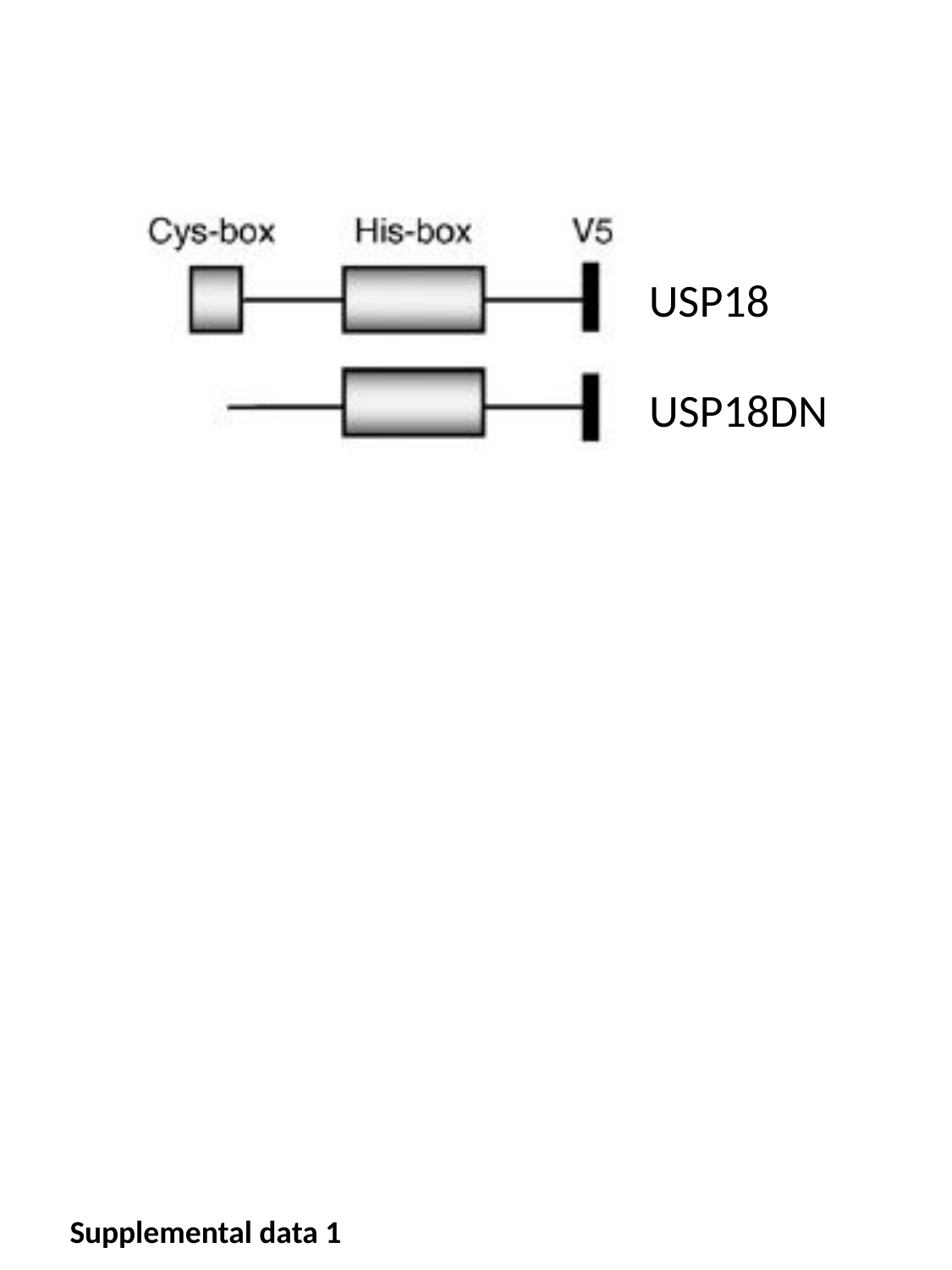

USP18
USP18DN
Supplemental data 1

Supplement: Supplementary file 1 [file mmc1.pptx]
